# Mycoplasma detection by triplex real-time PCR in bronchoalveolar lavage fluid from bovine respiratory disease complex cases

**DOI:** 10.1186/s12917-017-1023-6

**Published:** 2017-04-08

**Authors:** Jan B. W. J. Cornelissen, Freddy M. de Bree, Fimme J. van der Wal, Engbert A. Kooi, Miriam G. J. Koene, Alex Bossers, Bregtje Smid, Adriaan F. Antonis, Henk J. Wisselink

**Affiliations:** Department of Infection Biology, Central Veterinary Institute of Wageningen UR, P.O. Box 65, 8200 AB Lelystad, The Netherlands

**Keywords:** Bovine *Mycoplasma*, *M. dispar*, *M. bovis*, *M. bovirhinis*, Triplex PCR, RespoCheck, Bovine respiratory disease

## Abstract

**Background:**

In this study we evaluated the RespoCheck *Mycoplasma* triplex real-time PCR for the detection in bronchoalveolar lavage fluid (BALF) of *Mycoplasma (M.) dispar*, *M. bovis* and *M. bovirhinis*, all three associated with bovine respiratory disease (BRD). Primers and probes of the RespoCheck *Mycoplasma* triplex real-time PCR are based on the V3/V4 region of the 16S rRNA gene of the three *Mycoplasma* species.

**Results:**

The analytical sensitivity of the RespoCheck triplex real-time PCR was, as determined by spiking experiments of the *Mycoplasma* strains in Phosphate Buffered Saline, 300 colony forming units (cfu)/mL for *M. dispar*, and 30 cfu/mL for *M. bovis* or *M. bovirhinis*. The analytical sensitivity of the RespoCheck *Mycoplasma* triplex real-time PCRwas, as determined on purified DNA, 10 fg DNA per assay for *M. dispar* and 100 fg fo r*M. bovis* and *M. bovirhinis*. The analytical specificity of the RespoCheck *Mycoplasma* triplex real-time PCR was*,* as determined by testing *Mycoplasmas* strains (*n* = 17) and other bacterial strains (*n* = 107), 100, 98.2 and 99.1% for *M. bovis*, *M. dispar* and *M. bovirhinis* respectively. The RespoCheck Mycoplasma triplex real-time PCR was compared with the PCR/DGGE analysis for *M. bovis*, *M. dispar* and *M. bovirhinis* respectively by testing 44 BALF samples from calves.

**Conclusion:**

In conclusion, the RespoCheck PCR assay can be a valuable tool for timely and accurate detection of three *Mycoplasma* species associated with in bovine respiratory disease.

## Background

Bovine respiratory disease complex (BRDC) is a global problem causing severe economic losses to the cattle farming industry through mortality, loss of production, and treatment costs [1, 2]. It has a complex etiology that involves various pathogens, host factors, and environmental factors. Viruses such as bovine herpes 1 virus (BoHV-1, parainfluenza virus 3 (PBIV-3), bovine respiratory syncytial Virus (BRSV), respiratory bovine coronavirus (BoCoV) and bovine viral diarrhoea virus (BVDV) in conjunction with stress factors have been implicated as causes of respiratory tract infections of cattle by immunosuppression and damage to the respiratory epithelium [3]. A primary viral infection can be followed by an opportunistic secondary infection with bacteria like *Mannheimia haemolytica*, *Pasteurella multocida*, *Histophilus somni*, or *Trueperella pyogenes* [2, 4, 5], but these bacteria could also act as primary pathogen. In addition it has become increasingly clear that Mycoplasmas are important contributors to BRD, either as primary pathogens or in co-infection [2, 6–9]. *M. bovis* is the best known *Mycoplasma* species causing respiratory disease [4, 7], but also *M. dispar* and *M. bovirhinis* have been associated with BRD [2, 9–11]. *M. bovis* has not only been identified as a primary or opportunistic pathogen in BRD in beef cattle worldwide, but it has also been implicated in other clinical manifestations in cattle, such as mastitis, otitis, arthritis, and reproductive disorders [7]. *M. bovirhinis* and *M. dispar* are regularly isolated from the nasal cavity of cattle with respiratory disease and are usually regarded as an opportunistic pathogen in respiratory diseases [7, 12].

Bacteriological, serological and histopathological examinations are important tools to detect particular animal-carriers of *Mycoplasma* [13], however, these assays are time-consuming, insensitive and can give false positive results. Bronchoalveolar lavage fluid (BALF**)** from calves with BRD may contain various potential pathogens, but additional antibiotic use in the affected herds can inhibit cultivation and thereby can cause false-negative test results. In BRD, differential diagnosis of these pathogens with rapid turnaround time procedure is essential to implement appropriate treatment and intervention measures in a timely manner. Rapid detection of these pathogens at the early stage of outbreak can contribute substantially to minimize the spread of infection and increase treatment efficiency. Today quick, highly sensitive and species-specific PCRs are used in the diagnosis of *Mycoplasma*-associated diseases for *M. dispar* [14, 15], *M. bovis* [4, 16] and *M. bovirhinis* [17] in BALF or nasal swabs. Combining a 16S Ribosomal DNA PCR with denaturing gradient gel electrophoresis fingerprinting (PCR/DGGE) enabled the simultaneous detection of mixed *Mycoplasma* populations, however information about the detection limit in clinical samples is limited [18]. Additionally, a DNA microarray assay was developed for the parallel detection of 37 *Mycoplasma* species [19], in which species-specific probes derived from the 23S rRNA and *tuf* genes were used for species differentiation.

Multiplex real-time PCR could be a promising and practical approach to speed up the differential diagnosis from 1 to 2 weeks for traditional culture to 24 h, with limited expenses. This will make diagnostic testing more accessible for veterinary practitioners and thereby improve BRD diagnosis. This report describes the RespoCheck triplex PCR developed by Central Veterinary Institute (CVI, Lelystad, The Netherlands) for detection of three *Mycoplasma* species.

## Methods

### Strains and growth conditions

*M. bovis* (ATCC 25025) and *M. bovirhinis* (ATCC 5189985) were purchased from the ATCC (United Kingdom (U.K.), Guernsey, Ireland, Jersey and Liechtenstein) and cultured in Heart Infusion Broth Medium (Difco, Detroit, Mich.). All isolates were grown at 37 °C and 5% CO_2_ for seven days in a modified standard mycoplasma broth medium [20] containing 19 g of Heart Infusion Broth, 50 mL of liquid yeast extract (10% [vol/vol]; Oxoid, London, United Kingdom), 2 × 10^6^ U of penicillin G (Hoechst, Frankfurt, Germany), and 200 mL of heat-inactivated (56 °C, 30 min) horse serum per liter. Stocks of each isolate were prepared by freezing 1 mL portions of a 10 mL logarithmic-phase broth culture with 15% glycerol at −80 °C. Cultures were titrated on Heart Infusion Agar and were shown to contain 7 × 10^6^ cfu/mL for *M. bovis* and 4 × 10^5^ cfu/mL for *M. bovirhinis*. *M. dispar* NCTC 10125) was provided by Helena Windsor *(Mycoplasma* Experience LTD, Bletchingley, UK) with a titre of 1.6 × 10^7^ cfu/mL. In addition, DNA from 14 *Mycoplasma* strains (Table 1) were provided by Prof. Konrad Sachse (Friedrich-Loeffler-Institut, Federal Research Institute for Animal Health, Bundesforschungsinstitut für Tiergesundheit, Jena, Germany).

**Table 1:** *Mycoplasma* strains (*n* = 17), which were used as reference material

Species (Type strain)	ID	Ct-values		
		*M. bovis*	*M. dispar*	*M. bovirhinis*
*M. agalactiae (PG2)*	R 41^b^	20.9	-	-
*M. alkalescens PG 31/D 12*	R 18^b^	-	35.7^a^	-
*M. bovis PG45*	R 9^b^	19.4	-	-
*M. bovirhinis PG43*	R 12^b^	-	-	25.8
*M. bovigenitalium PG11*	R 8^b^	-	-	-
*M. californicum ST-6*	R 26^b^	-	-	-
*M. canadense 275C*	R 22^b^	-	-	-
*M. canis, PG14*	R 74^b^	-	-	20.0^a^
*M. dispar 462/2.*	R 11^b^	-	18.7	-
*M. leachii PG50* *(former M. bovine group VII)*	R 23^b^	-	-	-
*M. mycoides subsp. Mycoides PG1* *(former Small Colony Type)*	R 84^b^	-	-	-
*Acholeplasma axanthum S743*	R 17^b^	-	33.1^a^	-
*A. laidlawii PG8*	R 10^b^	-	-	-
*A. oculi 19-L*	R 62^b^	-	-	-
*M. bovis*	ATCC 25025	23.9	-	-
*M. dispar (NCTC 10125)*	ATCC 27140	-	20.3	-
*M. bovirhinis*	ATCC 5189985	-	-	20.9

^a^Cross-reactions in the RespoCheck triplex Mycoplasma PCR

^b^ID Friedrich-Loeffler-Institut

Hundred and seven bacterial isolates, representing 39 different species, were used to evaluate of specificity of the RespoCheck *Mycoplasma* triplex real-time PCR assay (Table 2). These included isolates associated with BRD and isolates associated with other bovine diseases. Prior to testing by PCR, the identity of the isolates was confirmed using MALDI-TOF mass spectrometry (MS Bruker MALDI Biotyper Microflex, version 3.1 with the reference database version 3.1.66 Bruker Daltonics GmbH, Germany).

**Table 2:** Bacterial strains (*n* = 107), that were used as reference material

Identification (number of isolates tested)	CCUG identification^c^	Source
*Acidovorax spp.* (3)	NA^c^	CVI collection^a^
*Actinomyces*	NA	CVI collection^a^
*Aerococcus viridans*	NA	CVI collection^b^
*Bibersteinia trehalosi*	*Pasteurella trehalosi*	CCUG 37711
*Biberstenia trehalosi* 20 AA III 3 E3	NA	CVI collection^a^
*Biberstenia trehalosi* 21 AA III 3 E4	NA	CVI collection^a^
*Brucella abortus*	NA	CVI collection^b^
*Comamonas kerstersii*	NA	CVI collection^a^
*Corynebacterium bovis (2)*	NA	CVI collection^b^
*Corynebacterium pseudotuberculosis*	NA	CVI collection^b^
*Escherichia coli*	NA	CVI collection^b^
*Gallibacterium anatis (5)*	NA	CVI collection^a^
*Hafnia alvei*	NA	CVI collection^a^
*Histophilus somni*	NA	ATCC 22132^e^
*Histophilus somni (4)*	NA	CVI collection^a^
*Klebsiella oxytoca*	NA	CVI collection^b^
*Klebsiella pneumoniae*	NA	CVI collection^b^
*Lactobacillus mucosae*	NA	CVI collection^a^
*Lactococcus garvieae*	NA	CVI collection^b^
*Lactococcus lactis*	NA	CVI collection^b^
*Listeria monocytogenes*	NA	CVI collection^b^
*Mannheimia heamolytica*	NA	ATCC 14003
*Mannheimia heamolytica*	NA	CVI collection^a^
*Mannheimia haemolytica*	*Mannheimia glucosida*	CCUG 38457-T
*Mannheimia granulomatis*	*Mannheimia granulomatis*	CCUG 45422-T
*Mannheimia granulomatis* 25 AA III 3 E8	NA	CVI collection^a^
*Mannheimia haemolytica*	*Mannheimia ruminalis*	CCUG 38470-T
*Mannheimia haemolytica (5)*	NA	CVI collection^a^
*Mannheimia haemolytica* 3 AA III 2 H2	NA	CVI collection^a^
*Mannheimia varigena*	*Mannheimia varigena*	CCUG 38462-T
*Mannheimia varigena* 19 AA III 3 E2	NA	CVI collection^a^
*Mannheimia varigena* 24 AA III 3 E7	NA	CVI collection^a^
*Micrococcus luteus*	NA	CVI collection^b^
*Moraxella bovis*	NA	CVI collection^b^
*Moraxelle lacunata* (2)	NA	CVI collection^a^
*Mycobacterium avium subsp. paratuberculosis*	NA	CVI collection^b^
*Mycobacterium bovis*	NA	CVI collection^b^
*Mycobacterium tuberculosis*	NA	CVI collection^b^
*Neisseria zoodegmatis*	NA	CVI collection^a^
*Pantoea agglomerans Erwina herbicola (n = 13)*	NA	CVI collection^a^
*Pasteurella multocida*	NA	ATCC 15743^e^
*Pasteurella multocida*	NA	CVI collection^a^
*Pasteurella multocida*	Bisgaard Taxon 13	CCUG 16497^d^
*Pasteurella multocida*	Bisgaard Taxon 13	CCUG 16498^d^
*Pasteurella multocida*	*Pasteurella multocida* ss *gallicida*	CCUG 17978-T^d^
*Pasteurella multocida*	*Pasteurella multocida* ss *septica*	CCUG 17977-T^d^
Not typable	*Pasteurella aerogenes*	CCUG 27905^d^
*Proteus mirabillis*	NA	CVI collection^a^
*Pseudomonas aeruginosa*	NA	CVI collection^b^
*Psychrobacter* spp.	NA	CVI collection^a^
*Salmonella enteritica* ssp. *enteritica* serovar Dublin	NA	CVI collection^b^
*Salmonella enteritica* ssp. e*nteritica* serovar Typhinurium	NA	CVI collection^b^
*Serratia marcescans*	NA	CVI collection^b^
*Staphylococcus aureus*	NA	CVI collection^b^
*Staphylococcus epidermidis*	NA	CVI collection^b^
*Streptococcus agalactiae*	NA	CVI collection^b^
*Streptococcus bovis* (5)	NA	CVI collection^a^
*Streptococcus dysgalactiae*	NA	CVI collection^b^
*Streptococcus faecalis*	NA	CVI collection^b^
*Streptococcus hyointestinalis*	NA	CVI collection^a^
*Streptococcus pluranimalium* (5)	NA	CVI collection^a^
*Streptococcus pneumoniae*	NA	CVI collection^b^
*Streptococcus* spp. (3)	NA	CVI collection^a^
*Streptococcus uberis*	NA	CVI collection^b^
*Trueperella pyogenes*	NA	ATCC 9731^e^
*Trueperella pyogenes (5)*	NA	CVI collection^a^
*Yersinia enterolytica*	NA	CVI collection^b^
*Yersinia pseudotuberculosis*	*Pasteurella lymphangitidis*	CCUG 27188-T^d^

^a^Isolated from lungs of calves

^b^Isolated from other tissues of cattle as lungs

^c^Not applicable

^d^CCUG: Culture Collection University of Götenborg, Sweden

^e^ATCC: American Type Culture Collection, USA

All bacterial strains, except the CCUG strains, were from an in-house strain collection

### Field samples and isolation of DNA

Calves (*n* = 44) with or without BRD (increased respiratory rate and/or dyspnoea) were sampled for diagnostic purposes. Sampling of the calves was granted an exemption from requiring ethics approval by the institutional Animal Experiment Commission “Dier Experimenten Commissie (DEC) Lelystad (2013111.b)” because sampling was performed for diagnostic purposes. BAL samples were obtained as described [21]. Approximately 35–75 ml BAL was obtained from each calf after instillation of 100 ml PBS with 10% Fetal Calf serum (FCS). Foam, large purulent exudates and blood clots were removed from the BALF samples under aseptic conditions. BALF (25 mL) was centrifuged (4600×g, 10 min, 4 °C). Sediment was resuspended in 0.5 mL Dulbecco’s minimal essential medium (DMEM) with 5% FCS, carefully added to 1 mL freeze medium (DMEM, 50% FCS and 20% DMSO) and frozen at −80 °C. The BALF supernatants were also stored at −80 °C.

For testing the influence of centrifugation of BALF samples (4600×g, 10 min, 4 °C) on the PCR results we tested three variants of BALF samples: without centrifugation, supernatant and pellet obtained after centrifugation (50 times concentrated). DNA was extracted from 200 μL aliquots of BALF samples. We used the MagNA Pure LC Total Nucleic Acid Isolation Kit (Roche Applied Science), with the Total NA External_lysis” protocol (Version 2.11). With the MagNA Pure LC Total Nucleic Acid Isolation Kit) 32 samples can processed per run. In all runs a positive control (a mix of 1.4 × 10^6^ cfu/mL *M. bovis,* 0.5 × 10^7^ cfu/mL *M dispar* and 1.3 × 10^5^ cfu/mL *M. bovirhinis*) and a negative water control (NTC) was included.

### RespoCheck primers and probes

To enable testing of testing for BRD associated pathogens in a routine setting, real-time PCRs for detection of viral, bacterial and mycoplasma pathogens in bronchoalveolar lavage fluid (BALF) of calves have been set up by the Central Veterinary Institute (Lelystad, The Netherlands) under the name RespoCheck. Primers and probes specific for the bacterial 16S, V3 and V4 regions were based on the Full length, bacterial 16S sequences (50,000 in July 2012) were used from the *nuccore* database at the National Center for Biotechnology Information (NCBI, USA, http://www.ncbi.nlm.nih.gov/nuccore). For *M. bovirhinis* and *M. dispar* the nearly full length 16S sequences were used. These sequences and their taxonomic information were used to build an Insignia-based database [22] from which pathogen-specific sequence regions were extracted with special interest for the V3 and V4 region because these sequences are often targeted for metagenomic next-generation sequencing (NGS) [23]. Using the identified regions, primers and probes were designed with AlleleID 7.8. (Premier Biosoft, palo Alto, USA). The resulting triplex PCR was designated RespoCheck *Mycoplasma* triplex real-time PCR The specificity of the *Mycoplasma* primers and probes was also verified against V3-V4 partial sequences of *M. flocculare*, *M. ovipneumonia* and *M. hyopneumonia.*

### RespoCheck triplex and single real-time PCR

The QuantiFast triplex Kit Real Time-PCR kit (Qiagen) was used for the RespoCheck *Mycoplasma* triplex real-time PCR. The assays were conducted in a 20 μl reaction mix containing 5 μl of the nucleic acid sample, 250 nM of each primer, 100 nM of each MGB probe, 1× QuantiFast triplex Real Time-PCR Master Mix and sterile deionised water. All reactions were conducted with an ABI-7500 with the following cycling parameters: 95 °C for 15 min, followed by 40 cycles of 94 °C for 15 s and 60 °C for 60 s. The machine was set to acquire fluorescence on the FAM, VIC, and NED channels for respectively *M. bovis, M. dispar and M. bovirhinis* All primers and probes were obtained from Life Technologies Europe BV (Bleiswijk, the Netherlands). The final results were analysed using ABI-7500 software (Version 1.4). Samples with a Ct of 40 cycles or less were considered to be positive.

### Evaluation of the analytical sensitivity and the analytical specificity

The analytical sensitivity of the RespoCheck triplex PCR was defined as the ability to detect the lowest concentration of *M. bovis, M. dispar* and *M. bovirhinis* expressed as a concentration (cfu/mL) [24]. The analytical sensitivity of the single and triplex PCRs for *M. bovis, M. dispar* and *M. bovirhinis* was determined with DNA isolated from 200 μL culture (*M. bovis, M. dispar* and *M. bovirhinis* strain) in a volume of 200 μL elution buffer at a final DNA concentration of 10 ng/ μL. This DNA preparation was tested in seven 10-fold serial dilutions (5 μL per assay) in PBS, resulting in a range with 10 ng down to 1 fg M*ycoplasma* DNA per assay. The Ct was determined for each sample by single and RespoCheck triplex real-time PCR with a threshold of 50% of the Delta Rn value (log). The threshold was manually set at 0.04 in the linear phase of the amplification plot, whereby the Slope and Correlation Coefficient values were 3.22 and 99.99% respectively.

The analytical sensitivity of the *M. bovis, M. dispar* and *M. bovirhinis* single and RespoCheck triplex real-time PCR, was also determined by testing a mixture of *M. bovis* (3 × 10^6^ cfu/mL), *M. dispar* (3 × 10^6^ cfu/mL) and *M. bovirhinis* (3 × 10^5^ cfu/mL) in seven 10-fold serial dilutions in BALF of specific pathogen free (SPF) calves of 3–4 weeks old. Dilution resulted in a series of *M. bovis*, *M. dispar* and *M. bovirhinis* spiked BALF samples, ranging from 3 × 10^6^ cfu/mL down to 0.3 cfu/mL. Total DNA was isolated from each 200 μl sample with the MAGNA pure isolation kit and the Ct was determined for each sample (5 μl) by both the single and RespoCheck triplex PCR assays. The slope of the curve, the efficiency and the detection limit (for DNA ng/μl; for cells cfu/mL) for each PCR was determined. To determine the analytical specificity of the designed RespoCheck triplex PCR, 17 *Mycoplasma* isolates and 107 bacterial strains (Table 2) were tested.

### Diagnostic sensitivity and specificity in BALF samples from calves.

For determining the diagnostic specificity, BALF samples were analysed with the PCR/DGGE method by the Animal and Plant Health Agency (APHA, Mycoplasma Team, Addlestone Surrey, UK) as earlier described [18, 25]. To determine the analytical sensitivity of the PCR/DGGE analysis, four 10-fold serial dilutions of *M. bovis* (7 × 10^4^ cfu/mL), *M. dispar* (16 × 10^4^ cfu/mL), and *M. bovirhinis* (0.5 × 10^4^ cfu/mL), were prepared in PBS. Samples were sent to the APHA and analysed using the PCR/DGGE method.

### Sequencing amplicons

16S rDNA PCR-sequencing was used for confirmation of the results of RespoCheck *Mycoplasma* triplex real-time PCR. 16S rDNA of the DGGE positive /PCR positive (*n* = 5) and DGGE negative /PCR positive (*n* = 5) was amplified using the specific *Mycoplasma* primers of the RespoCheck *Mycoplasma* triplex real-time PCR. DNA was sequenced by BaseClear (Leiden, the Netherlands) by an automated DNA sequencer. The nucleotide sequences were compared with GenBank sequences using the Basic Local-Alignment Search Tool(BLAST) of the NCBI-NIH for homology [26]. Pair-wise sequence alignments were performed using the Clustal algorithm implemented in the program DNA star (DNASTAR Inc., Madison, WI).

### Analyses of sensitivity and specificity

The analytical sensitivity of the RespoCheck triplex PCR was determined by its ability to detect a low concentration of *M. bovis, M. dispar* and *M. bovirhinis* and therefore expressed as a concentration (ng/assay and cfu/mL) [24]. The analytical specificity of the assay was calculated for each target microorganism using the following definition for specificity as the percentage of true negative samples/ the number of true negative samples and the number of false positive samples [27].

Calculation of diagnostic sensitivity, specificity and Cohen’s Kappa Coefficient was performed as described [28]. We therefore used the results of the PCR/DGGE analysis as reference standard.

### Statistical analyses

Differences in PCR results were analysed for statistical significance by the non-parametric Mann–Whitney U test in the GraphPad Prism version 5.0 software, with *P* < 0.05 considered significant.

## Results

### Analytical sensitivity and linear detection range of the RespoCheck triplex

**Fig. 1:**
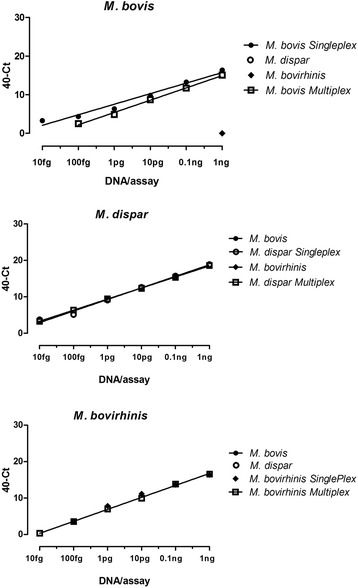
Analytical sensitivity of *M. bovis*, *M. dispar* and *M. bovirhinis* in single and RespoCheck triplex PCR assays. Ten-fold serial dilutions of *M. bovis*, *M. dispar* and *M. bovirhinis* DNA were made in PBS in a range from 1 ng down to 10 fg/assay. The resulting samples were tested in the *M. bovis*, *M. dispar* and *M. bovirhinis* single and RespoCheck triplex real-time PCR

**Fig. 2:**
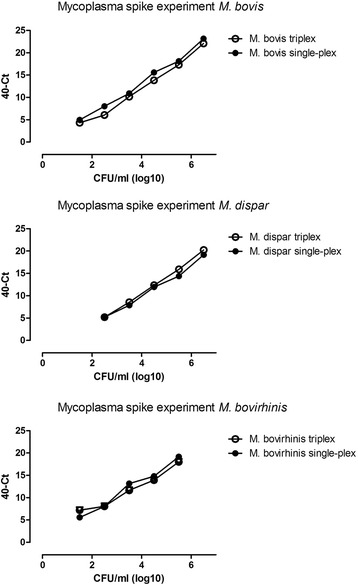
Analytical sensitivity of *M. bovis*, *M. dispar* and *M. bovirhinis* single and RespoCheck triplex real-time PCR in spiked BALF samples. Ten-fold serial dilutions of *M. bovis*, *M. dispar* and *M. bovirhinis* were made in BALF samples in a range from 3 × 10^6^ down to 0.3 cfu/mL. The resulting samples were subjected to DNA isolation and testing in the *M. bovis*, *M. dispar* and *M. bovirhinis* single and RespoCheck triplex real-time PCR

The linearity of quantification of the RespoCheck triplex *Mycoplasma* real-time PCR was established through a linear regression plot by plotting the Ct-values against the values of log10 DNA concentration tested per reaction. The *M. dispar* single and RespoCheck triplex real-time PCR showed a linear detection range from 10 ng to 10 fg DNA per assay with a linear correlation (R^2^) value of 0.999 (Table 3; Fig. 1.). The *M. bovis* and *M. bovirhinis* single and RespoCheck real-time PCR showed a linear detection range from from 1 ng to 100 fg DNA per assay, with a R^2^ value of 0.999 (Table 3; Fig. 1). In BALF spiked samples, the detection limit of the RespoCheck triplex real-time PCR was 300 cfu/mL for *M. dispar*, and 30 cfu/mL for *M. bovis* or *M. bovirhinis* (Table 3﻿; Fig. 2). In the RespoCheck *Mycoplasma* real-time PCR, 5 μL was tested and the analytical sensitivity was therefore 1–15 cfu/assay. A good linear correlation (R^2^ > 0.96) was found between the values of BALF spiked samples and the Ct-values in the RespoCheck *Mycoplasma* triplex or singleplex real-time PCR for the three Mycoplasmas (Table 3).

**Table 3:** Performance of the RespoCheck *Mycoplasma* real-time PCR in which the *M. dispar, M. bovis* and *M. bovirhinis* DNAs were diluted in PBS (A) or cells were spiked in BALF (B)

A						
PCR	Agent	Real time PCR				
		R^2^	Slope	Efficiency (%)	Linearity (ng)	Detection limit (ng/assay)
Singleplex PCR	*M. dispar*	0.9995	−3.3836	97.49	10 ng-10 fg	10 fg
	*M. bovis*	0.9966	−3.1137	109.49	10 ng-10 fg	10 fg
	*M. bovirhinis*	0.9955	−3.5033	92.95	10 ng-10 fg	10 fg
Triplex PCR	*M. dispar*	0.9989	−3.1175	109.3	10 ng-10 fg	10 fg
	*M. bovis*	0.9955	−3.6240	88.8	10 ng-100 fg	100 fg
	*M. bovirhinis*	0.9939	−3.3735	97.9	10 ng-100 fg	100 fg
B						
PCR	Agent	Real time PCR				
		R^2^	Slope	Efficiency (%)	Linearity (CFU/ml; log 10)	Detection limit (CFU/assay)
Singleplex PCR	*M. dispar*	0.995	3.248 ± 0.1276	103.2	6.5–2.5	1–2
	*M. bovis*	0.995	3.453 ± 0.1178	94.8	6.5–1.5	0.5
	*M. bovirhinis*	0.981	3.395 ± 0.2698	97.0	5.5–1.5	0.5
Triplex PCR	*M. dispar*	1.000	3.534 ± 0.04608	91.9	6.5–2.5	1–2
	*M. bovis*	0.993	3.462 ± 0.1440	94.5	6.5–1.5	0.5
	*M. bovirhinis*	0.965	2.750 ± 0.3014	131.0	5.5–1.5	0.5

**Fig. 3:**
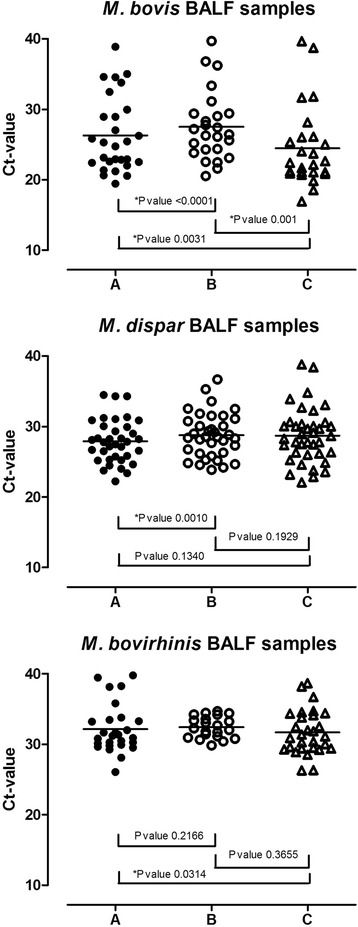
Ct-values obtained in the RespoCheck *Mycoplasama* triplex real-time PCR on DNA samples derived from BALF samples from *M. bovis*, *M. dispar and M. bovirhinis* infected calves. PCRs were performed on three variants of BALF samples: without centrifugation (A), supernatant after centrifugation (B) and sediment of after centrifugation (50 times concentrated) (C)

**Fig. 4:**
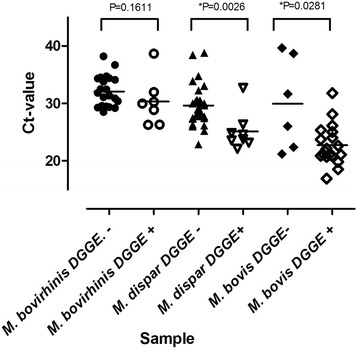
The Ct-level of DNA derived from BALF samples from *M. bovis*, *M. dispar* and *M. bovirhinis* infected calves of PCR/DGGE analyses of DGGE APHA negative and positive samples. Significant *P* values are indicated by *

**Fig. 5:**
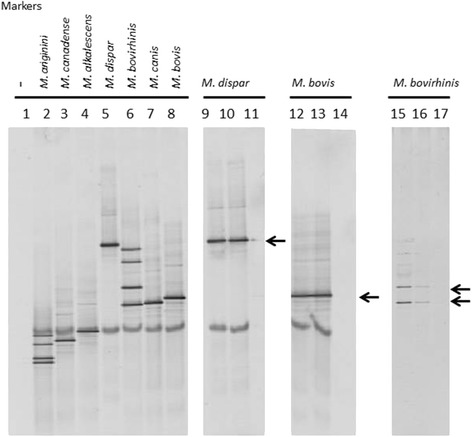
DGGE fingerprinting profiles of 16S ribosomal DNA fragments obtained after amplification by PCR. Lane 1 contains the negative water control, lanes 2 to 8 contain several Mycoplasma strains as reference (lane and strain designations indicated), lanes 9, 10 and 11 contain three 10-fold serial dilutions of *M. dispar* (starting with 16 × 10^4^ cfu/mL), lanes 12, 13 and 14 contain three 10-fold serial dilutions of *M. bovis* (starting with 7 × 10^3^ cfu/mL) and lanes 15, 16 and 17 contain three 10-fold serial dilution of *M. bovirhinis* (starting with 0.5 × 10^4^ cfu/mL)

### Analytical specificity of the RespoCheck triplex PCR

RespoCheck Mycoplasma triplex PCR in silico BLAST searchs (http://blast.ncbi.nlm.nih.gov/Blast.cgi) for the specificity of the *M. dispar* amplicon revealed a 100% identity (E-value 1E^−46^) for 2 hits for *M. dispar* complete genome sequence. The in silico BLAST search for amplicon of *M. bovis* we found a 99–100% identity (E-values 4E^−43^ - 6E^−45^) to 31 complete genome or 16S ribosomal partial sequence, and for the amplicon of *M. bovirhinis* we found a 99–100% identity (E-values 5E^−50^ - 1E^-51.^) For the *M. bovirhinis* amplicon a 97% identity (*3E-40*) was found for 11 hits for *Mycoplasma canis* (Taxid:29,555).

Seventeen *Mycoplasma* strains (Table 1) and 107 bacterial strains (Table 2) were used to calculate the analytical specificity of the RespoCheck *Mycoplasma* triplex PCR*.* The RespoCheck *Mycoplasma* triplex real-time assay for detecting *M. bovis, M. dispar* and *M. bovirhinis* possessed an analytical specificity of 100% (0 FP), 98.2% (2 FP) and 99.1% (1 FP), respectively. No cross-reactivity in the RespoCheck *Mycoplasma* triplex real-time assay was observed with any of the 107 bacterial strains. The *M. dispar* PCR did however cross-react with *Acholeplasma axanthum* S743 and *M. alkalescens* PG 31/D 12. In silico sequence data analyses from the 16S V3 genomic DNA region showed no similarity of *M. dispar* specific sequences with the *A. axanthum* S743 and *M. alkalescens* PG 31/D 12 isolates. The *M. bovirhinis* RespoCheck triplex PCR cross-reacted with *M. canis* with a Ct-value of 20.4. In the *M. bovis* RespoCheck triplex PCR we found a cross-reaction with *M. agalactiae*. Based on the almost 100% similarity of *M. canis* PG14 16S rRNA gene and the *M. bovirhinis*16S rRNA, it is not possible to prevent for this cross-reaction.

### Diagnostic sensitivity and specificity of the RespoCheck triplex compared with DGGE

To study the influence of centrifugation of the BALF samples on the PCR results we compared the PCR results from the BALF samples before and after centrifugation (10 min at 4600×g). A significant lower Ct-value (*P* < 0.05; non-parametric Wilcoxon statistics) in the RespoCheck *Mycoplasma* triplex real-time PCR was found for *M. bovis* and *M. dispar* in the pellet of the centrifuged BALF samples. Several *M. bovis*, *M. bovirhinis* and *M. dispar* mix-infections could be detected in one BALF sample with a difference of 10 Ct-values between the three species and were in accordance with the PCR/DGGE analysis (Fig 3). Therefore we used the pellet of the centrifuged BALF samples (50× concentrated) to determine the presence of the three *Mycoplasma* species in 44 BALF samples by real-time PCR.

The calculated diagnostic sensitivity and specificity the RespoCheck triplex PCR is reported in Table 4. As the diagnostic specificity is very low (0.1944, 0.739, 0.3889 for *M. dispar*, *M. bovis* and *M. bovirhinis* respectively) we analysed the sequence of the produced amplicon of five DGGE negative /PCR positive and five DGGE positive PCR positive samples. The sequence of both products was confirmed as *M. bovis*, *M. dispar* or *M. bovirhinis*, as all sequences had a high E-value (3e-44) and 100% Query cover (100%) against the homologue sequence using the BLAST of the NCBI-NIH. Comparison of the Ct-values of PCR positive/ DGGE negative and the PCR positive/ DGGE positive samples with a non-parametric Mann Whitney test, showed that the Ct values of *M. dispar* and *M. bovis* were significantly lower, *p* = 0.0026 and 0.0282, respectively. In the *M. bovis* and *M. dispar* PCR, the difference in Ct value between PCR positive/ DGGE positive and PCR positive/ DGGE negative samples is at least 3.2, which indicates a factor of 10 difference in concentration of *M. bovis* and *M. dispar* DNA between these two groups (Fig. 4). As a consequence the diagnostic specificity of the RespoCheck triplex PCR is undervalued by this method. We compared the results of the *M. bovis*, *M. dispar* and *M. bovirhinis* RespoCheck triplex PCR with the results of the PCR/DGGE analysis. The detection limit of the *M. bovis, M. dispar* and *M. bovirhinis* PCR/DGGE analysis was, as determined by APHA, 0.7 × 10^3^ cfu/mL, 16 × 10^3^ cfu/mL and 0.5 × 10^3^ cfu/mL, respectively (Fig. 5).

**Table 4:** Diagnostic sensitivity and specificity of the RespoCheck *Mycoplasma* triplex real-time PCR compared with the PCR/DGGE method

*M. dispar*	PCR +	PCR -	Total	Diagnostic specificity and sensitivity
DGGE +	8	0	8	Sensitivity =1
DGGE -	29	7	36	Specificity =0.1944 (95% CI: 0.0819–0.3602)
Total	37	7	44	
*M. bovis*	PCR +	PCR -	Total	Diagnostic specificity and sensitivity
DGGE +	20	1	21	Sensitivity =0.9524 (95% CI: 0.7618–0.9988)
DGGE -	6	17	23	Specificity =0.7391 (95% CI: 0.5159–0.8977)
Total	26	18	44	
*M. bovirhinis*	PCR +	PCR -	Total	Diagnostic specificity and sensitivity
DGGE +	7	1	8	Sensitivity =0.8750 (95% CI: 0.4735–0.9968)
DGGE -	22	14	36	Specificity =0.3889 (95% CI: 0.2314–0.5654)
Total	29	15	44	

## Discussion

PCR assays for the detection of *Mycoplasmas* generally target sequences on the 16S rRNA gene [29, 30]. In this study we used the highly conserved 16S rRNA sequence to set up the RespoCheck *Mycoplasma* triplex real-time PCR assay for the specific detection of *M. bovis*, *M. dispar* and *M. bovirhinis* in BALF samples of calves.

The lowest concentration of *M. dispar* which could be detected with the RespoCheck triplex PCR assay is around 300 cfu/mL. With a copy number of 16S rRNA of one or two (https://rrndb.umms.med.umich.edu/) and with a test volume of 5 μl the lowest concentration which could be detected is around 1–2 cfu/assay. The lowest concentration of *M. bovis* and M. *bovirhinis* which could be detected with the RespoCheck triplex for *M. bovis*, and *M. bovirhinis* is around 0.5 cfu/assay. From the calculated analytical sensitivity of the *M. bovis*, *M dispar* and *M. bovirhinis* RespoCheck triplex PCR (0.5–2 cfu/assay) we conclude that the RespoCheck triplex PCR has a good analytical sensitivity. It was shown that the use of a pellet from 25 mL BALF after centrifugation instead of not-centrifuged BALF samples increased the analytical sensitivity of the RespoCheck triplex PCR assay. In order to determine the analytical specificity of the RespoCheck triplex PCR we analysed the DNAs from panels of *Mycoplasma* and bacterial strains. In the *M. bovis* RespoCheck *Mycoplasma* triplex real-time PCR we found a cross-reaction with *M. agalactiae.* Phylogenetic analyses on 16S rRNA sequences and comparing the 16S rRNA sequences of *M. bovis* and *M. agalactiae* [25] at NCBI (www.ncbi.nlm.nih.gov), we found a close relationship between *M. agalactiae* and *M. bovis*, with a 99% nucleotide identity between their 16S rRNA sequences. However, *M. bovis* causes calf pneumonia, mastitis, and arthritis in cattle [16, 31], *M. agalactiae* is the causal agent of contagious agalactia in goats and sheep [32]. Although unusual, *M. agalactiae* has been detected from cattle samples [33, 34]. Therefore the cross reactivity for *M. agalactiae* might be a problem for the intended BALF samples in the *M. bovis* PCR. In the *M. bovirhinis* RespoCheck triplex real-time PCR one false positive reaction was obtained on DNA from *M. canis. M. canis* can be isolated from the reproductive tract of dogs, but has not been proved to cause disease in dogs. However, it has been shown to cause clinical signs of pneumonia in experimentally challenged calves [35] and *M. canis* has been isolated from ruminants in Britain [36, 37]. Depending on the incidence of *M. canis* in ruminants, this may give false-positive results in the *M. bovirhinis* RespoCheck triplex real-time PCR. DNA samples from *M. alkalescens* and *A. axanthun* showed high Ct-values (>35) for *M. dispar* in the RespoCheck triplex real-time PCR*,* and were therefore classified as false-positive (Ct of 40 cycles or less were considered to be positive). *M. alkalescens* and *M. bovigenitalium* are important *Mycoplasmas* that can infect cattle and cause mastitis, arthritis and respiratory disease [17]. However, in the sequence analyses of the PCR-positive and DGGE-negative *M. dispar* BALF samples, we did not find any indication for the presence of *M. alkascens*, underlining the high specificity for *M. dispar* in the RespoCheck triplex real-time PCR.

Monitoring for Mycoplasma species in BALF samples through collection and testing of BALF samples by culture is hampered by the fastidious nutritional requirements, lengthy culture of mycoplasmas, and their susceptibility to growth inhibitors. As a consequence, Mycoplasma culture is time-consuming, costly, and requires specific expertise. Moreover, Mycoplasma species may easily be overgrown by bacterial contaminants or by more rapidly growing *Mollicutes,* notably *Acholeplasmas*. The PCR/DGGE method of the APHA can differentiate 13 bovine *Mycoplasma* species [18] including the target Mycoplasmas of the RespoCheck *Mycoplasma* triplex real-time PCR and in contrary to the RespoCheck can differentiate between *M. bovis* and *M. canis*. Additional the PCR/DGGE is capable of detecting mixed cultures, which would have been difficult to detect by culture methods [18]. Therefore we used this method as a reference for determining the diagnostic sensitivity and specificity of the RespoCheck *Mycoplasma* triplex real-time PCR.

Possibly due to the lower sensitivity of the DGGE analysis compared to the RespoCheck triplex PCR (almost factor 10) and its use as reference method to validate the RespoCheck triplex PCR, the latter test method scores 29, 6 and 22 *M. dispar*, *M. bovis* and *M. bovirhinis* respectively out of 44 more samples as false-positive and therefore the diagnostic specificity of the RespoCheck triplex PCR is underestimated. The transport and storage conditions or differences in DNA preparation of particularly the more diluted BALF samples for the PCR/DGGE method could have induced a lower sensitivity of the PCR/DGGE analysis. The Ct values of the *M. bovis*, and *M. dispar* PCR positive and DGGE positive samples are significant (*P* < 0.05 Mann Whitney test) lower than the *M. bovis*, and *M. dispar* PCR positive DGGE negative samples, which confirms the difference in the analytical sensitivity between the RespoCheck triplex PCR and DGGE analyse. In the *M. bovis* and *M. dispar* PCR, we found a 10 fold difference in the Ct values between the DGGE positive/ PCR positive and DGGE negative/ PCR positive samples, which indicates a higher diagnostic sensitivity of *M. bovis* and *M. dispar* PCR than the DGGE analyses. Results by DGGE from BALF samples with mixed infections could be reproduced by the triplex PCR, suggesting that there is no significant PCR bias when the triplex PCR is used for Mycoplasma detection in field samples. The PCR has thus a higher analytical sensitivity than the DGGE.

## Conclusion

In conclusion, the RespoCheck *Mycoplasma* triplex PCR-test appears to be a sensitive and specific test for the detection of *M. bovis*, *M. dispar* and *M. bovirhinis* in BALF samples of calves.

## Abbreviations

BALF: Bronchoalveolar lavage fluid
BRDC: Bovine respiratory disease complex
PCR/DGGE: PCR with denaturing gradient gel electrophoresis fingerprinting
